# Antidiabetic Potential of *Mangifera indica* L. cv. Anwar Ratol Leaves: Medicinal Application of Food Wastes

**DOI:** 10.3390/medicina55070353

**Published:** 2019-07-09

**Authors:** Mohammad Saleem, Muiz Tanvir, Muhammad Furqan Akhtar, Mazhar Iqbal, Ammara Saleem

**Affiliations:** 1 Punjab University College of Pharmacy, University of the Punjab, Lahore 54000, Pakistan; 2 Department of Pharmacology, Faculty of Pharmaceutical Sciences, Government College University Faisalabad, Faisalabad 38000, Pakistan; 3 Riphah Institute of Pharmaceutical Sciences, Riphah International University, Lahore Campus, Lahore 54000, Pakistan; 4 Health Biotechnology Division, National Institute for Biotechnology and Genetic Engineering (NIBGE), Jhang Road, Faisalabad 38000, Pakistan

**Keywords:** anwar ratol, mango, mass spectrometry, mangiferin, flavonoids, postprandial glucose

## Abstract

*Background and objectives:* Anwar Ratol is one of the most famous cultivar of mango in South Asia, especially Pakistan. Mango leaves are left as food waste. This study evaluated the potential of mango (Anwar Ratol) leaves for their use against diabetes mellitus. *Material and Methods:* In this study, hydro-alcoholic extract of the plant leaves was prepared and evaluated by electrospray ionization mass spectroscopy (ESI-MS) and high-performance liquid chromatography (HPLC) for the presence of phytochemicals. The plant extract was administered to Alloxan induced diabetic mice followed by evaluation through oral glucose tolerance test; determination of postprandial glucose, body weight, lipid profile and histopathological evaluation of pancreas. *Results:* Chemical evaluation revealed the presence of mangiferin, rhamnetin, catechin, epicatechin, iriflophenone 3-C-β-D-glucoside, gallic acid and other phenolic and flavonoid compounds. The plant extract exhibited a decrease in postprandial blood glucose following seven days therapy in diabetic mice. The extract also prevented the rise in blood glucose level as determined by glucose tolerance test in diabetic mice. Furthermore, therapy of diabetic mice with the extract prevented a decrease in body weight and decline in beta-cell mass associated with alloxan and improved lipid profile. *Conclusion:* The findings of the study clearly suggested that the leaf extract of the plant might possess anti-diabetic activity possibly due to the presence of mangiferin and other phytochemicals such as phenolic and flavonoid compounds. This study will serve as a basis for the use of mango leaf extract against diabetes. Furthermore, this study will also provide basis for the bioassay-based fractionation and isolation of active principles responsible for the antidiabetic potential of mango leaves.

## 1. Introduction

Diabetes mellitus (DM) is a chronic metabolic disease. Antidiabetic medicines lack rigorous control on DM and exhibit different troublesome adverse effects. Therefore, medicinal plants and food wastes are explored for pronounced antidiabetic activity and less severe adverse effects [1,2].

The plant, *Mangifera indica* L. cultivar Anwar Ratol, commonly known for its sweetness, belongs to Ancardiaceae family. The genus *Mangifera* contains 69 species of which less than half of the plants produce edible fruits. The mango plant bark is traditionally used to treat diarrhea, cancer, diabetes, prostatitis, toothache and cough and urinary tract and skin infections. The stem bark is also used as emetic, diuretic, antiseptic, astringent and hepatoprotective agent [3]. Studies have shown that the stem bark exhibited anti-inflammatory and anti-amoebic properties, prevented DNA damage and lipid peroxidation in rats and showed immunomodulatory and analgesic properties. Leaf extracts have shown hepatoprotective, antiulcerogenic, hypolipidemic, antioxidant and antibacterial activity against both gram positive and negative microorganisms [4,5]. Peel, stem bark and leaf extracts have shown hypoglycemic activity in diabetic rats. Plant seeds have also shown antibacterial activity. Several compounds isolated from stem bark, leaf and fruits include mangiferin, rhamnetin glycoside, quercetin and kaempferol O-glycoside, Indicoside A and B, manghopanal [4,6].

The mango plant extracts have shown in vitro and in vivo antioxidant activities [5]. The plant extracts contain flavonoids and polyphenols. Various parts of this plant have also shown hypoglycemic activity [4]. However, very little is known about its Pakistani cultivar, Anwar Ratol, which is one of the most popular varieties of mango in South Asia. Therefore, this study focused on the phytochemical and anti-diabetic potential of the leaves of *M. indica* cultivar Ratol in albino mice.

## 2. Material and Methods

Alloxan monohydrate and standard analytical grade chemicals were obtained from Sigma Aldrich^®^, Merck (Taufkirchen, Germany). Glibenclamide was acquired from Sanofi Aventis (Karachi, Pakistan). Crescent diagnostic kits^®^ were used for serum cholesterol, high density lipoprotein (HDL), low density lipoprotein (LDL) and triglycerides. HPLC grade solvents were purchased from Merck, Kenilworth, NJ, USA). Accuchek performa Glucometer was purchased from Roche Diagnostics GmbH^®^ (Mannheim, Germany) to measure blood glucose. Syringes were acquired from Becton Dickinson^®^, BD (Franklin Lakes, NJ, USA). The mass spectrometer used was LTQ XL (Thermo Electron Corporation^®^, Waltham, MA, USA).

### 2.1. Collection and Extraction of the Plant

The young leaves (current year) of *Mangifera indica* L. cultivar Anwar Ratol were obtained from a private mango garden in Multan. The plant leaves were identified by a taxonomist at University of Peshawar, and a specimen was deposited under voucher no. Bot.20152 (PUP). Damaged leaves and dust were removed followed by shade drying. Then the leaves were crushed and reduced to a coarse powder with a domestic grinder. Hydro-alcoholic mixture was selected as solvent based on literature review so as to extract polar phytochemicals such as phenolics [7]. The powder was macerated in a hydro-alcoholic mixture (30:70) for 7 days with occasional shaking. The extract was filtered with Whatman filter paper and dried with a rotary evaporator. The resulting concentrated extract was refrigerated in an amber colored glass bottle for further use.

### 2.2. Mass Spectrometry

The qualitative phytochemical analysis was performed by both Mass spectrometry (mass-mass analysis) and HPLC. For mass spectrometry, the sheath gas flow rate was set at 25 L/min while auxiliary gas flow rate was 5 L/min. Capillary voltage and temperature were –20 V and 300 °C respectively. The sample was analyzed at negative scan with the mass spectra range of m/z 100–1000. Helium gas was used for collision. The flow rate of the sample was 0.3 mL/min [8,9]. Mobile phases comprised of 2% acetic acid (A) and acetonitrile (B) with a gradient program: 5% (10 min), 10% (1 min), 40% (9 min), 20% (10 min), 40% (10 min) and 100% acetonitrile until the completion of the run. Collision induced spectra was obtained in the range of 1.2–1.7 V fragmentation amplitude [8]. Phytochemicals were identified from the NIST library while comparing their mass spectra and retention times.

### 2.3. Quantification of Phenolic and Flavonoid Compounds

The HPLC analysis was performed using Shimadzu shim-pack CLC-ODS (C-18) column. Two mobile phases, A (H_2_O: Acetic acid 94:6 at pH = 2.27) and B (100% acetonitrile), were run at 1 mL/min flow rate. UV-Vis detection was done at 280 nm. The peaks and retention times were compared to those of standards [7].

### 2.4. Experimental Animals

Swiss albino mice of either sex weighing 25–30 g were used in the experiment. The mice used were born and bred in the Animal house of GC University Faisalabad. The animal study was approved by Institutional Review Board, GC University Faisalabad on 10 June 2016 with Reference number GCUF/ERC/2016/1108. The animals were kept at a closely maintained temperature of 25 ± 2 °C with a relative humidity of 44–56%. Mice were kept under a 12 h light and dark cycle. The animals were provided with standard rodent pellet diet and water ad libitum.

### 2.5. Induction of Diabetes

Diabetes was chemically induced by intraperitoneal injection of alloxan monohydrate at 150 mg/kg dose. Before alloxan injection, mice were fasted overnight. The mice were monitored for 4 days for blood glucose level. Mice with a blood glucose level ≥200 mg/dL were selected to be used in the study [8].

The mice were divided into six groups with 6 mice in each group. Group 1 served as a normal control group and contained normal animals. Group 2 was a standard drug control group receiving 10 mg/kg glibenclamide. Group 3 was disease control. Other groups received 550, 750 or 950 mg/kg plant extract.

### 2.6. Oral Glucose Tolerance Test

For oral glucose tolerance test in diabetic mice, each group received its respective dose of either the extract or the standard drug followed by glucose solution 2 g/kg after 30 min. The blood glucose level was monitored at 0, 30, 60 and 120 min following glucose administration [8].

### 2.7. Effect on Postprandial Blood Glucose Level

The postprandial blood glucose level was measured 2 h after the first meal of day, given to mice immediately after the administration of plant extract or standard drug. Normal control mice received distilled water only. The blood was obtained from the tail to monitor changes in glucose levels [9].

### 2.8. Biochemical Parameters and Histopathology

The mice were anesthetized with chloroform after 7 days therapy with the extract. The blood was collected and serum was separated for the determination of biochemical parameters such as cholesterol, serum triglyceride, HDL and LDL with commercial kits. The pancreas, liver and kidney of mice were carefully removed and preserved in 10% formaldehyde. Tissue sections were prepared with microtome and stained with Hematoxylin and eosin dyes so as to be observed under a light microscope [10,11].

### 2.9. Statistical Analysis

The data were analyzed using GraphPad Prism^®^ software version 5.01 (GraphPad Software Inc., San Diego, CA, USA). Results were expressed as mean ± standard error. Two-way ANOVA was applied to analyze the data of blood glucose level and weight variation in mice. One-way ANOVA was applied to evaluate the effect of hydro-alcoholic extract on lipid profile in mice.

## 3. Results

### 3.1. Phytochemical Analysis

Phytochemical analysis was carried out by ESI-MS and HPLC. Chemical evaluation carried out by ESI-MS revealed different phytochemicals such as rhamnetin, catechin, epicatechin, gallic acid derivatives, mangiferin and iriflophenone 3-C-β-D-glucoside. Detected phytochemicals, their molecular formula and mass to charge ratio are shown in Table 1.

Chemical entities were identified on the basis of literature review and confirmed via ESI-MS analysis [12,13,14,15,16,17]. Meanwhile HPLC analysis showed the presence of gallic acid, ferulic acid, *m*-coumaric acid, quercetin, chlorogenic acid and benzoic acid in the extract. Chlorogenic acid was present in the maximum amount followed by benzoic acid in the extract as shown in Table 2.

### 3.2. Effect on Oral Glucose Tolerance and Postprandial Blood Glucose

**Figure 1:**
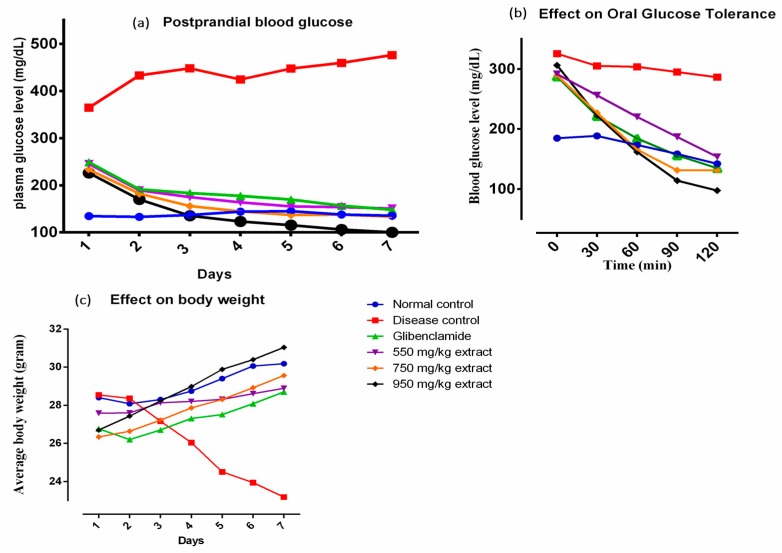
Effect of *M. indica* cultivar Anwar Ratol leaf extract on (**a**) postprandial blood glucose in diabetic mice; (**b**) oral glucose tolerance test in diabetic mice; (**c**) body weight of diabetic mice.

Intake of plant extracts significantly prevented the rise in blood glucose level than the disease control group, 2 h following the administration of glucose solution (Figure 1b). Furthermore, a therapy for seven days with 550, 750 or 950 mg/kg extract in diabetic mice showed remarkable decrease in postprandial blood glucose level compared to untreated diabetic mice. The postprandial blood glucose level in experimental groups was comparable to the glibenclamide treated mice (*p* ˂ 0.05) (Figure 1a).

### 3.3. Effect on Body Weight and Lipid Profile of Diabetic Mice

**Figure 2:**
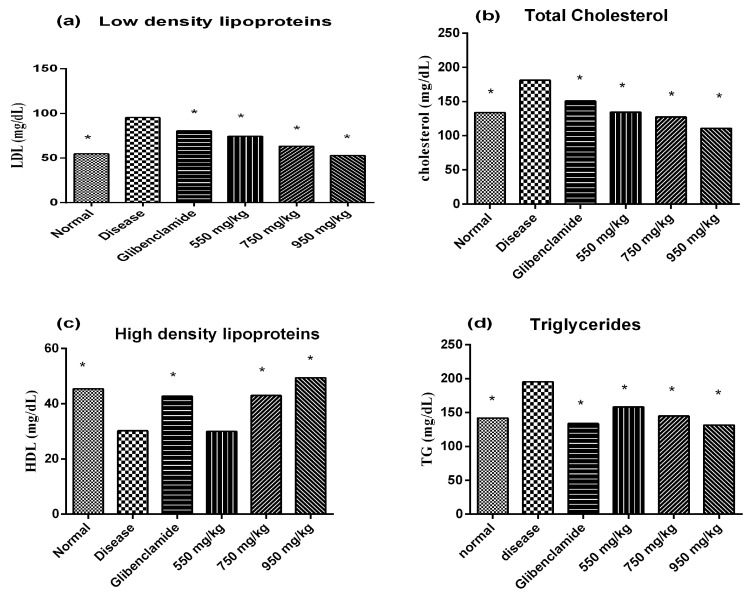
Effect of *M. indica* cultivar Anwar Ratol extract on lipid profile of diabetic mice over a period of one week. Results were presented as mean ± S.D (*n* = 6) and analyzed by one-way ANOVA. * represented significantly different as compared to untreated diabetic control group (*p* < 0.001). Where LDL: low density lipoprotein; HDL: high density lipoprotein.

Increase in body weight of diabetic mice was observed at all dosage levels. Positive effect on body weight was observed within 5 days of starting therapy with the plant extract (Figure 1c). Treatment with the plant extract or glibenclamide ameliorated the lipid profile in alloxan treated mice. The results clearly showed that the extract significantly lowered low density lipoproteins (LDL), cholesterol and triglycerides and raised high density lipoproteins (HDL) dose dependently in diabetic mice compared to the untreated diabetic mice (Figure 2).

### 3.4. Effect on the Histology of Pancreas, Kidney and Liver

**Figure 3:**
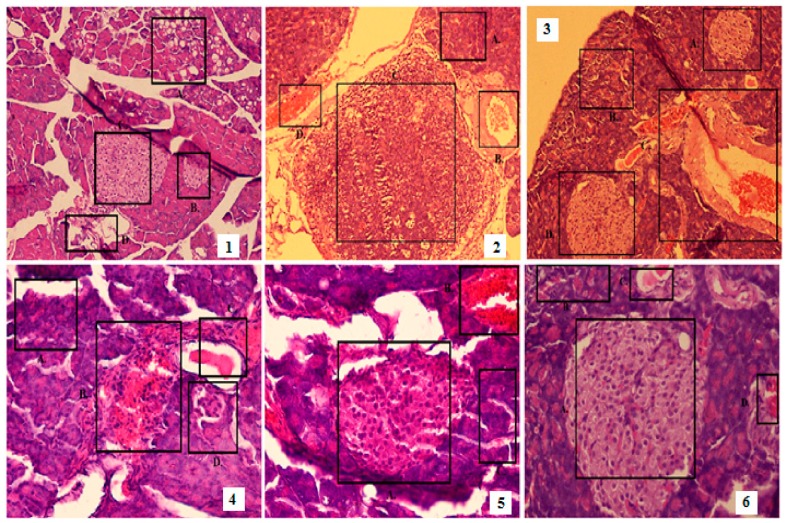
Photomicrographs of diabetic mice pancreas treated with *Mangifera indica* extract. Where Islets of langerhan are shown by boxes in normal, disease control, diabetic mice treated with glibenclamide, 550, 750 and 950 mg/kg extract (shown by 1–6 respectively).

The plant extract showed a protective effect on the pancreas of diabetic mice. Treatment with the extract decreased the damage to beta cells and acinii and restored endocrine and exocrine pancreatic structure. It also prevented inflammation and congestion of the veins in diabetic mice. The diseased group showed complete destruction of the islets compared to the standard and experimental groups (Figure 3).

**Figure 4:**
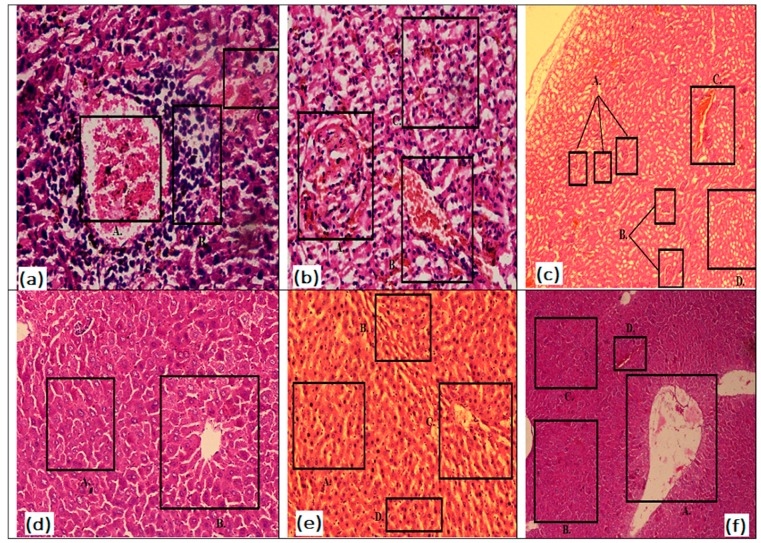
Photomicrographs of diabetic mice kidney and liver treated with *Mangifera indica* extract. Here, disease control kidney, standard therapy kidney, 950 mg/kg extract treated rat kidney, normal control liver, liver of diabetic mice treated with 550 and 950 mg/kg extracts are shown by (**a–f**) respectively. Where glomeruli are shown by boxes in (**a**), (**b**) and (**c**), whereas boxes show normal hepatocytes in (**d**), pyknosis in (**e**) and central vein in (**f**) respectively.

Histopathological study of the kidney in disease control mice showed the chronic infiltrations of inflammatory cells such as lymphocytes and plasma cells as evidenced along with hemorrhage around the damaged glomerulus. Treatment with glibenclamide partially reversed the glomerular damage with some loss of cellularity and focal damage to the kidney. Standard control mice also showed normal tubules with congested blood vessels. Treatment with the extract improved the histology of diabetic mice dose dependently. It was seen that the treatment with plant extract at 950 mg/kg showed an intact glomeruli, normal tubules and blood vessels (Figure 4a–c).

Histopathology of disease control mice showed the damaged hepatocytes with condensation of chromatin (pyknosis). Diabetic mice treated with the plant extract at 550 mg/kg resulted in normal hepatocytes around central vein; however, pyknosis was present in the liver. Diabetic mice treated with 950 mg/kg plant extract showed normal liver histology and minimal damage to hepatocytes as compared to animals treated with 550 and 750 mg/kg extract dose (Figure 4d–f).

## 4. Discussion

In this study, hydro-alcoholic extract of mango leaves was evaluated by mass spectrometry and HPLC for the presence of various phytochemicals such as phenolic, flavonoid and alkaloid compounds. The plant extract was administered to diabetic mice to demonstrate its effect on oral glucose tolerance, postprandial glucose level, biochemical parameters, body weight and histopathological changes in the pancreas of diabetic mice.

The HPLC and GC-MS analysis revealed the presence of mangiferin, rhamnetin, catechin, epicatechin, iriflophenone 3-C-β-D-glucoside, gallic acid derivatives and other phenolic and flavonoid compounds. These phytochemicals have been reported previously in mango plant and fruits. Mangiferin is the main antioxidant present in the plant, mainly useful in cardiovascular and neurodegenerative diseases [18]. These phenolic and flavonoid compounds have been commonly reported in fruits, kernel, leaves and stem bark of other mango cultivars [4].

Intake of plant extracts significantly prevented the rise in blood glucose in the disease control group 2 h before the administering glucose solution. Furthermore, the plant extract prevented the rise in the fasting blood glucose in diabetic rats. Various phytochemicals present in mango leaves are thought to be responsible for its anti-hyperglycemia activity. Previously, it was shown that foliamangiferosides such as mangiferin had exerted their antidiabetic effect through increasing insulin sensitivity and inhibiting alpha-glucosidase activity [6]. Iriflophenone 3-C-β-D-glucoside has also been reported to exhibit anti-diabetic potential [19]. Previous studies have demonstrated that the inhibition of postprandial glucose and fat utilization of the body were attributed to chlorogenic acid. Chlorogenic acid also decreased LDL level and increased fat combustion. Furthermore, polyphenol rich extract of coffee had also been linked to the inhibition of glucagon like peptide [20]. Gallic acid was found to induce GLUT 4 and thus increased glucose uptake. It was also found that phenolic compounds inhibited a rise in blood glucose concentration by blocking alpha glucosidase and pancreatic lipase activities [21]. Quercetin also possesses antidiabetic activity through preventing damage to the pancreas and ameliorating endogenous antioxidant enzymes [6]. Therefore, it can be speculated that mangiferin, iriflophenone 3-C-β-D-glucoside, quercetin and other polyphenols present in the plant leaves may be responsible for hypoglycemic activity of the extract.

This study revealed that the administration of plant extract ameliorated the lipid changes induced by alloxan in diabetic mice. Previously, it was found that catechin, epicatechin, chlorogenic acid, gallic acid and mangiferin were responsible for decreasing hyperlipidemia in diabetic animals. A lipid lowering activity of the plant extract may be associated with the presence of these phytochemicals [7,22,23].

Histopathological study showed that the plant extract had exhibited a protective effect on the pancreas of diabetic mice via decreasing the damage to beta cells and acini and restoring the pancreatic structure. Previously, it was shown that mangiferin had improved beta-cell regeneration in rats. Quercetin also reduced alloxan induced damage to beta cells. Ferulic acid and gallic acid also prevented beta-cell damage through a decrease in oxidative stress [24].

## 5. Conclusion

The present study clearly showed that the hydroalcoholic extract of *M. indica* cultivar Anwar Ratol leaves contained mangiferin and iriflophenone 3-C-β-D-glucoside and phenolic and flavonoid compounds. The plant extract may possess considerable antidiabetic activity as shown by a decrease in postprandial blood glucose level. Furthermore, it increased glucose tolerance, body weight, improved lipid profile and decreased the damage to beta cells. Therefore, the leaf extract of the plant may be useful in managing diabetes and its complications such as weight loss and alteration in lipid profile.

## Figures and Tables

**Table 1:** Phytochemicals detected in *Mangifera indica* cultivar Anwar Ratol by mass spectrometry analysis.

Sr. No.	Compound Name	Molecular Formula	MS-MS [M–H]−	Major Fragment Ions (m/z, %)
1	Iriflophenone 3-C-β-D-glucoside	C_19_H_21_O_10_	407.2	317 (27), 287 (100), 245 (3)
2	Iriflophenone 3-C-(2-O-hydroxybenzoyl)-β-D-glucoside	C_26_H_24_O_11_	559.1	439 (2), 407 (96), 389 (11), 317 (21), 287 (100), 269 (61), 245 (13), 169 (32)
3	Mangiferin	C_19_H_18_O_11_	421.1	403 (7), 331 (86), 301 (100), 271 (8)
4	Gallic acid	C_7_H_6_O_5_	169.1	125 (100)
5	Methyl gallate	C_8_H_8_O_5_	183.1	168 (15), 124 (100)
6	Tetra-O-galoyl glucoside	C_34_H_28_O_22_	787.0	635 (39), 617 (100), 465 (37)
7	Penta-O-galoyll glucoside	C_41_H_32_O_26_	939.1	787 (12), 769 (100), 617 (41)
8	Ellagic acid	C_14_H_6_O_8_	301.1	300 (62), 283 (56), 245 (20), 200 (55), 145 (100)
9	Epicatechin	C_15_H_14_O_6_	289.0	Not yet reported
10	Digalloyl glucose	C_20_H_20_O_11_	483	Not yet reported
11	Rhamnetin	C_16_H_12_O_7_	315	300 (26), 193 (37), 165 (100)
12	Ethyl gallate	C_9_H_10_O_5_	197.0	Not yet reported
13	Tri-O-galloyl glucoside	C_27_H_24_O_18_	635	Not yet reported

**Table 2:** Phenolic and flavonoids determined by HPLC analysis in hydro-alcoholic extract of *Mangifera indica* cultivar Anwar Ratol.

Compound Name	Retention Time	Area (mV.s)	Area (%)	Concentration (ppm)
Quercetin	3.200	20.488	1.6	1.08
Gallic acid	4.380	83.019	6.4	2.98
Benzoic acid	14.987	15.847	1.2	1.67
Chlorogenic acid	15.373	48.032	3.7	3.79
*m*-coumaric acid	20.447	15.632	1.2	0.18
Ferulic acid	22.467	36.627	2.8	2.63

## Supplementary Files

Supplementary File 1
