# Supplementary material for: Antidiabetic Potential of Mangifera indica L. cv. Anwar Ratol Leaves: Medicinal Application of Food Wastes
Source: Medicina (Kaunas). 2019 Jul 9;55(7):353. doi: 10.3390/medicina55070353 (PMC6681213; doi:10.3390/medicina55070353)
Supplement: Supplementary File 1 [file medicina-55-00353-s001.pdf]

Supplementary Material

Article

Antidiabetic Potential of *Mangifera indica* L. cv. Anwar Ratol Leaves: Medicinal Application of Food Wastes

Mohammad Saleem [1,*], Muiz Tanvir [2], Muhammad Furqan Akhtar [3,*], Mazhar Iqbal [4] and Ammara Saleem [2]

[1] Punjab University College of Pharmacy, University of the Punjab, Lahore, Pakistan
[2] Department of Pharmacology, Faculty of Pharmaceutical Sciences, Government College University Faisalabad, Faisalabad, Pakistan, muiztanvir@gmail.com (M.T), amarafurqan786@hotmail.com (A.S)
[3] Riphah Institute of Pharmaceutical Sciences, Riphah International University, Lahore Campus, Lahore, Pakistan
[4] Department of Health Biotechnology, NIBGE, Jhang Road, Faisalabad, Pakistan migondal@nibge.org (M.I)
* Correspondence: saleem2978@hotmail.com (M.S.), furqan.pharmacist@gmail.com (M.F.A.)

ESI-MS Spectrum of *Mangifera indica* leaf extract

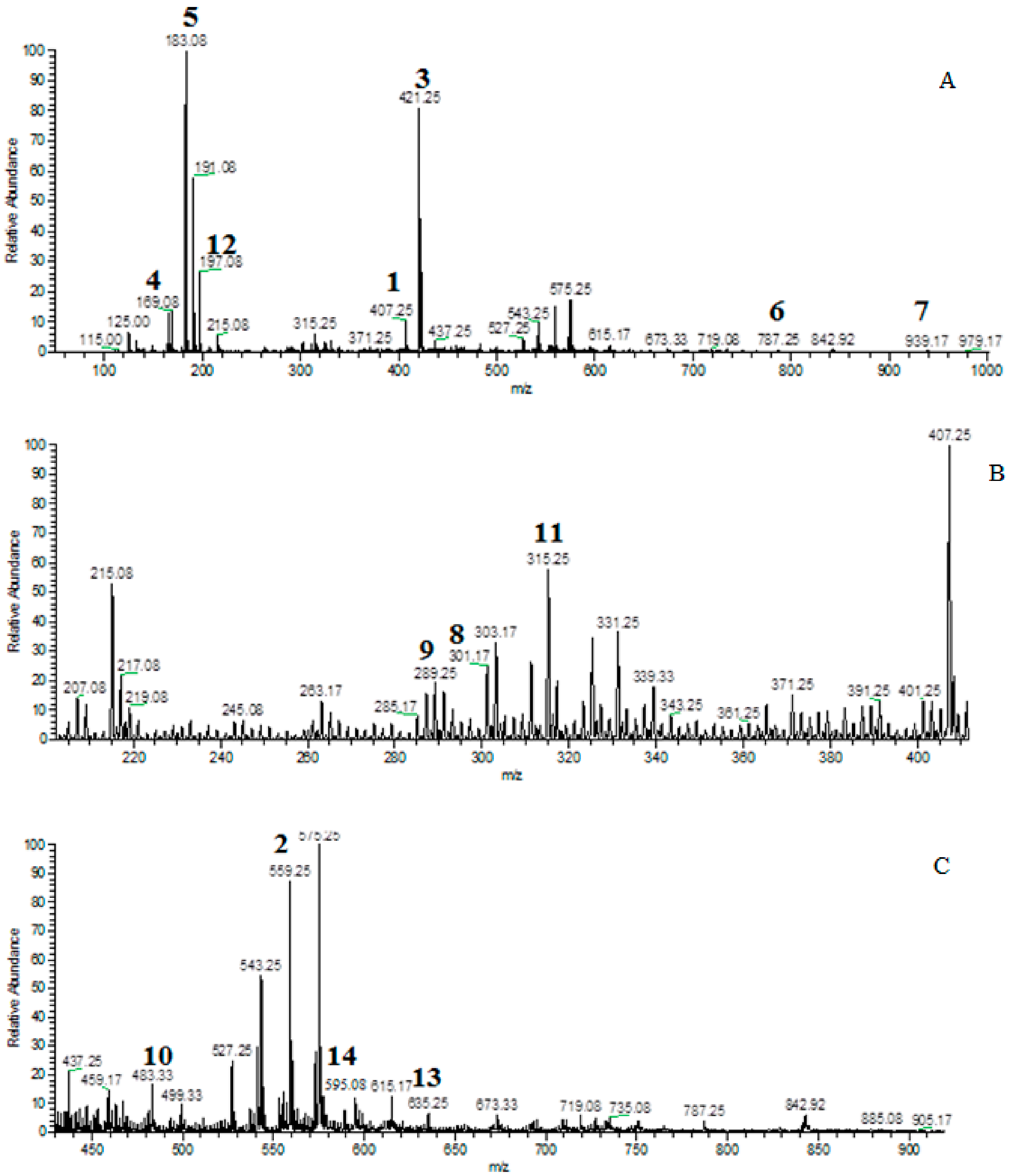

Figure S1. Magnification of peak between the mass ranges of 100–1000, 200–450 and 450–900 m/z.
